# On the Use of Large Doses of Quinine

**Published:** 1845-06

**Authors:** 


*On the use of large doses of Quinine.—Report of a Committee of the Medical Department of the National Institute, on Dr. Buck's Paper, " On the Use and Abuse of Medicine."*—Your Committee, to whom was referred the paper of Marcus C. Buck, M.D., beg leave to present the following as their views on the subject of his communication.

The title of the paper is " On the Use and Abuse of Medicine." In treating of this subject, the writer has taken some very sensible and proper views. In the commencement of the paper, the author avows the object he had in view in writing and reading it before the National Institute. The object is a most laudable one, and with the views entertained by the author, he could not conscientiously refrain from this course. Humanity, as well as the interests of society, required it of him. We are also pleased to observe the spirit of tolerance and the courteous and complimentary style of the paper. Although he attacks the doctrine and practice of a very large portion of the surgeons of the Army, he extends to them, individually, and to the head of the corps particularly, well-deserved compliments.

The object of Dr. Buck's paper, is to oppose his observations and experience, to the practice resorted to within the last few years, of giving large doses of quinine, and to disapprove of its use. Many very cogent and substantial arguments are introduced by him, to sustain his motto, " *In medio, tutissimus ibis.*"

In order that the committee may be fully understood in the views and opinions they have expressed, it will be proper to pass in review, cursorily, the subject and object of the paper to which Dr. Buck's is intended as an offset or corrective.

This paper was read before the convention of the National Institute by Surgeon Van Buren, U. S. A. This, if we understand the nature of the paper correctly (not having heard it read,) was to present, in a concise form, the evidences of the advantages of large single doses of quinine, over small and repeated doses of the medicine, in malarial diseases. And these evidences were drawn directly from recorded materials presented to the Medical Bureau of the U. S. A., by medical men of the highest standing, the most unimpeachable veracity, and after their frequent and repeated trials of the medicine in their practice in the South. If what I have heard of the nature of this paper be true, no opinion was expressed by the compiler, and no theory was deduced from the facts. His paper was a mere statement of facts collected in the manner above stated, and elicited by the Surgeon General, with the view to the more correct understanding of the subject. He did elicit full and important details, which go far to prove that the medicine may be given in large doses with impunity, and with a decided medical effect. The facts presented by this paper of Dr. V. B. cannot, or rather should not, be compared to those detailed by Dr. Buck, of the administration of large doses of opium and other poisons with advantage, because we know that 99-100ths of persons would be killed by this indiscriminate opium practice. Whether or not that would be the effect of quinine, is shown by repeated observations made by army and other practitioners. It has been clearly shown, that no case of death, which has occurred after the use of large doses of quinine, has ever been traceable to this medicine; therefore his illustrations of the impropriety of this practice will not hold good.

In presenting his views on the subject, Dr. Buck has lost sight of a very important fact. The number of cases in which single and very large doses of quinine have been given, is so great, that it no longer produces a query, whether this can be done. Upwards of two thousand cases have been treated in Florida in this way, and with far greater success than ever was known to arise from any other method, and, as I have said, without death resulting from it. There has as much as one ounce of sulphate of quinine been given, and no ill effects occur from it.

Dr. Buck takes the ground, that if small doses will have the desired effect, why resort to such large doses? This, of course, is a most sensible view of the matter. *If small doses will have the* desired effect, why resort to so large a dose? What has been the experience of all those who have used this medicine in large and small doses? They all tell you that it required larger doses in Florida to produce a certain effeet; that the small doses, repeated, as was wont to be our practice up to this era, proved inefficient. They tell you, moreover, that small doses repeated, do not produce the same decided and permanent impression as the large single dose; that small doses produce more certain and decided cerebral derangement than the single large dose. Now these are not theories, not mere speculation, but the result of actual observation. Thus it is shown by these gentlemen, that in several cases of delicate females and others, when small doses of the medicine were given, severe consequences followed; while the administration of one full dose, of 20 or 30 grains, so far from producing cerebral disturbance, had the contrary effect. This, I say, is not theory, but fact; and many such facts are on record as coming from army surgeons and several private practitioners, both in Florida and other malarial countries. It is true that there are a few rare instances of blindness and deafness being produced by this wholesale practice; but have we not known the same to occur from the continued use of quinine in small doses? Therefore the few cases in which unpleasant effects have arisen from the use of this medicine, should not weigh, when we have thousands in this country alone of the decided beneficial effects of it. Idiosyncracy may influence the action of this medicine, as in the case of Dr. Buck's opium-eaters and others. But we need not confine our remarks to the experience of surgeons in this country, as to the good effects of large doses of quinine in malarial diseases. We find in Europe that they are resorting to it. Among others, I will merely allude to the opinion of Dr. Austin Flint, of Buffalo, N. Y. (vide page 277, Vol. II. of the American Journal of Medicine.) And what has been the experience in this city and the surrounding country? Let me refer you to a paper by Dr. ———, of Maryland. There we find the administration of large doses of quinine advocated. And the same practice is followed, I hear, in the surrounding and adjacent counties of both Virginia and Maryland. In our own city we have the evidence of our friend Dr. Sewall, who has given it in drachm doses. (See London Lancet.)

Now, without going further, this is a weight of authority which Dr. Buck cannot disregard, nor can the medical world. It has been more than five years since Mr. Piorry first made his observations on the subject of giving large doses of quinine in enlarged spleen; and we must regard the accounts we have received of his success. But prior to his observations, and long before the Florida war, eminent men in this part of the country gave large doses of quinine. Dr. Potter gave 8 grains; and advocates of his precepts and practice have been increasing since he wrote and lectured on the subject. Let us, then, sum up the facts on this subject, for from these much may be argued on which to form the judgment.

In the first place, it has been shown by more than 2000 observations in this country, that large doses of from 10 to 60 grains, or an ounce, of quinine, can be given without producing injury.

2. That it has been proved, beyond doubt, that these large doses do exert a curative effect on periodical and malarial diseases, and more certainly than small doses.

3. That the cases of permanent injury resulting from large doses of quinine, are not more, indeed not so numerous, as from repeated small doses.

4. That the temporary inconvenience of disturbance of the nervous system is not so liable to ensue from large as small doses. This is stated, though our experience is to the contrary, in most cases.

5. That so far from smaller doses being more certain, they are not, the paroxysm being far more likely to occur after their use, than after a single large dose.

6. That the impression made on the system is more permanent from large than small doses.

7. That in diseases that run their course rapidly to a fatal termination, as in the southern country, a reliance on small doses was found to prove hazardous to the safety of the patient; therefore, when it is desirable to cut short or prevent the occurrence of a violent chill, the large doses should be resorted to.

8. The visceral diseases are not more liable to follow, if as much so, from large as from small doses of quinine.

Now these various conclusions, if true—and how can we doubt their truth, coming from the source they do?—should, to say the least, cause us to reflect before we denounce the practice of giving large doses of quinine, and call it rash and empirical.

Our *own* individual experience, though limited, is yet to us worth something. We have been in the habit, from long usage and from impressions imbibed, like Dr. B.'s, from lectures heard in our pupilage, of considering a grain or two repeated quite enough; and we feared to administer a full dose until within the last year, since when we have been in the habit of giving 10 and 15 grain doses, the night prior to the expected paroxysms of fever, and must say have seldom been disappointed with its effects. Convalescence succeeded to its administration, and seldom have we found it necessary to repeat the dose.

In the use of all medicines it is important and proper that we should discover the medicinal dose. More than this is of course superfluous. This is an important point to ascertain in the medicine now under consideration. And it should be the duty and determination of all who regard the interests of the community, and the science of medicine, to aid in the furtherance of this object. All over a certain amount is either inert or injurious. Our observations led us to consider that about 15 grains in this climate may be considered the *medium* dose, and that as much benefit will result from this dose as from two scruples or a drachm. Less than these doses will scarcely act as an anti-periodic medicine; but this dose, given at a proper period of time from the anticipated attack, will most certainly have the desired effect.

The next question to be ascertained, is, how long before the expected paroxysm should this dose be given? This is a very important fact to have fully ascertained, for it is an object to give it as far distant from the paroxysm as possible, for reasons well known to all who have ever used the medicine. We have found twelve hours answer exceedingly well, and this is the usual period of time allowed. But some recent observations have gone to prove that the anti-periodical effects are more decidedly felt eighteen hours after its administration.

Let us next ascertain in what class of diseases quinine is most suited, and whether we can account for the difference in the size of the dose, which has been given at different periods of time since its discovery.

The first part of this inquiry can readily be answered, if our opinion of the mode of the action of this medicine be true. We look upon it as purely an anti-periodic medicine, and indicated in all this class of diseases particularly, possessing peculiar medicinal virtues in malarial diseases. We hold that there is no purely tonic properties in quinine. We can readily conceive, therefore, that its action may be prejudicial, whether given in large or small doses, in diseases having an origin independent of malaria and not periodical in their type. Hence we find that Britteneau, Recamier, and others quoted by Dr. Buck, found it not only injurious but actually fatal in cases of this class. But it does not clearly appear, in the observations of these gentlemen, that death was more the result of the large doses of quinine which they administered than of the disease in which it was given. We should not be precipitate in referring death to the medicine administered in diseases which are so often fatal, particularly under the treatment of the French pathologists. And we should be careful, also, in ascertaining the effects of medicine on dogs and other animals, how far the action on these is applicable to the human economy. For it is a well-known fact, that many articles of the Materia Medica, which exert a baneful influence on the lower orders of animals, are not only innocuous to man, but possess a curative and sanatory effect on him. We have ever regretted that this mode of deducing the effects of medicine on the human economy should have been practised.

The question now arises, whether causes do not exist, *why* this medicine can be exhibited at the present day in larger doses than formerly. We entertain the idea that causes do exist for this. And among the *first*, we shall notice the *deterioration of the article.* This will account, in some degree, for the capability of the system to bear a larger dose than formerly. When this medicine was first discovered and introduced into practice, *one* grain was equivalent to one drachm of the best Peruvian bark. The medicine then sold for from $10 to $15, even $30 per ounce. What the proportionate dose of it is now, we are unable to say. But the price is now reduced to from $2,50 to $4,00 per ounce. Inasmuch as bark continues much the same in price, we would infer that there is nothing to justify the marked reduction of the price, unless it be the adulteration of the article, or the more slovenly mode of preparing it. The greater facility of making it, would of course reduce the price greatly. We then include this among the reasons why the system will bear larger doses, and why larger doses are required to produce the desired effect than formerly, though we do not by any means wish to be understood to assert that the system would not formerly have borne larger doses than were then given.

*(To be continued.)*

